# Telehealth Delivery of Speech–Language Pathology Services for Children with Cleft Palate and Velopharyngeal Dysfunction: A Systematic Review

**DOI:** 10.3390/children12111523

**Published:** 2025-11-11

**Authors:** Nisreen Naser Al Awaji, Alanoud Nawaf Alsinan, Raja S. Alamri, Nourah A. Bin Ruaydan, Lama S. Alharbi, Lana T. Albesher, Latifa Alrutaiq

**Affiliations:** Department of Health Communication Sciences, College of Health and Rehabilitation Sciences, Princess Nourah bint Abdulrahman University, P.O. Box 84428, Riyadh 11671, Saudi Arabia

**Keywords:** cleft palate, velopharyngeal dysfunction, telehealth, speech–language pathology, pediatrics, PRISMA

## Abstract

Background/Objectives: This systematic review aimed to evaluate the effectiveness, caregiver satisfaction, and accessibility of telehealth-delivered speech–language pathology (SLP) services for children with cleft palate and/or velopharyngeal dysfunction (VPD). Methods: Based on PRISMA 2020, we searched PubMed, CINAHL, Scopus, PsycINFO, and the Cochrane Library (2000–31 May 2025) for studies enrolling participants ≤ 18 years of age with cleft/VPD who received telehealth services (assessment, therapy, counseling/follow-up), with or without in-person comparators. Screening and data extraction were performed in duplicate. Risk of bias was appraised using RoB 2 (randomized) and CASP checklists (non-randomized/service designs). To account for heterogeneity, we conducted a SWiM-aligned narrative synthesis and summarized certainty with GRADE. Results: Eleven studies met the inclusion criteria. Telehealth delivery of SLP services was feasible and generally acceptable to families. Caregiver-mediated interventions frequently showed within-group improvements in speech outcomes, while remote assessment demonstrated moderate agreement with in-person ratings. However, the overall certainty of evidence was rated as very low to low because of small sample sizes, single-center designs, and heterogeneous outcomes. Conclusions: Telehealth is a feasible and acceptable mode for delivering SLP in pediatric cleft/VPD patients, with encouraging signals for caregiver-mediated articulation therapy and maintaining multidisciplinary follow-up. Implementation is best embedded within hybrid pathways, reserving in-person visits for complex assessments. Adequately powered comparative studies with standardized outcomes, longer follow-up, and equity-focused implementation are needed.

## 1. Introduction

Children born with cleft palate and/or velopharyngeal dysfunction (VPD) frequently present with persistent speech sound disorders arising from structural constraints on oral air pressure. These cleft speech characteristics (CSCs) are typically described as passive errors, reflecting structural insufficiency with generally correct placement but impaired manner, active errors, and learned compensations such as glottal stops or pharyngeal fricatives that shift the place of articulation [1,2,3]. Passive CSCs typically require surgical/structural management, whereas active CSCs respond to behavioral speech interventions delivered by a speech–language pathologist (SLP). Even with timely surgery and early therapy, some children continue to experience reduced intelligibility into school age, with downstream academic and psychosocial impacts [4,5]. Sustained, multidisciplinary care, including longitudinal SLP input within cleft team pathways, is therefore standard practice [6], in line with the American Cleft Palate–Craniofacial Association (ACPA) parameters for evaluation and treatment of patients with cleft lip/palate [7].

Access to high-quality, specialized SLPs for treating cleft/VPD remains uneven. Families in rural or underserved areas face barriers related to travel distance, limited clinician availability, scheduling constraints, and cost [8]. Telehealth offers an alternative mode for assessment, therapy, counseling, and follow-up. Across pediatric SLP more broadly, reviews have reported that telehealth can be clinically effective and acceptable when programs are structured and caregivers are engaged [9,10,11]. In principle, these features map well onto cleft/VPD care, which often relies on repeated articulation/phonology practice, caregiver coaching, and multidisciplinary review.

However, the cleft/VPD-specific evidence base for telehealth remains comparatively limited and methodologically heterogeneous. Key uncertainties include the following: (i) determining which services translate well to remote delivery (e.g., caregiver-mediated articulation/phonology therapy vs. perceptual assessment of resonance and airflow); (ii) the magnitude and consistency of clinical effects in this population relative to in-person care; (iii) caregiver satisfaction and engagement under real-world constraints; (iv) equity and feasibility in settings affected by bandwidth, device access, or language barriers [6,12,13]. Against this backdrop, telehealth is widely promoted to reduce travel burden and ensure multidisciplinary coordination, but robust, cleft-specific syntheses are sparse [14,15,16].

In response to the global acceleration of telehealth adoption during and following the COVID-19 pandemic, this review focuses specifically on SLP services within cleft and velopharyngeal dysfunction care, an area distinct from broader surgical or medical teleconsultations. Despite increasing clinical use, the evidence for telehealth’s effectiveness remains limited and methodologically heterogeneous, and broader reviews have highlighted persistent implementation and equity barriers influencing telehealth uptake and sustainability [17,18]. Accordingly, this review will evaluate the effectiveness, caregiver satisfaction, and accessibility of telehealth-delivered SLP services for this population.

Aim and Objectives

This systematic review aimed to evaluate the effectiveness, caregiver satisfaction, and accessibility of telehealth-delivered speech-language pathology (SLP) services for children with cleft palate and/or velopharyngeal dysfunction (VPD).

Objectives: (1) synthesize evidence across service types (assessment, therapy, and counselling/follow-up); (2) appraise study quality and risk of bias; (3) summarize implications for clinical practice and research within post-pandemic hybrid cleft care models.

## 2. Materials and Methods

### 2.1. Protocol and Registration

This review followed PRISMA 2020 guidelines [19]. The protocol was not prospectively registered, which is acknowledged as a limitation in terms of methodological transparency and discussed in Section 4.4. The final database search was performed on 31 May 2025. Any deviations from the initial plan (e.g., adding SWiM reporting) are disclosed herein. An internal review protocol outlining objectives, eligibility criteria, and synthesis procedures was drafted prior to database searching but was not formally registered in PROSPERO.

### 2.2. Eligibility Criteria (PICOS)

The eligibility criteria for this study were as follows:

Population (P): Children and adolescents (≤18 years) with cleft palate and/or VPD.

Interventions (I): Telehealth/telepractice delivery of SLP services (assessment, therapy, counseling, or follow-up) relevant to cleft/VPD.

Comparators (C): In-person care or usual care where available; studies without a comparator were also eligible if outcomes were reported.

Outcomes (O): (a) Effectiveness (e.g., articulation/phonology measures such as PCC, intelligibility, resonance/nasality ratings, validated speech outcomes); (b) caregiver/patient satisfaction; (c) accessibility/feasibility (e.g., attendance, travel/time saved, technology access, equity barriers).

Study designs (S): Randomized controlled trials (RCTs), non-randomized comparative studies, pre–post single-arm studies, retrospective cohorts, and analytical cross-sectional studies were considered. Purely qualitative studies were considered as adjunct evidence only.

Exclusions: Adult-only samples; non-SLP teleconsultations (e.g., general dentistry/restorative visits), unless they reported cleft/VPD-relevant SLP outcomes; editorials/letters, conference abstracts without data, and theses; studies lacking relevant outcomes; non-English publications.

Both comparative and single-group study designs were eligible. This inclusive approach was adopted because only a limited number of randomized or controlled studies exist in this field, and single-arm or service-evaluation reports provide valuable preliminary evidence on feasibility and within-group improvement. Findings on effectiveness derived from uncontrolled designs were, therefore, interpreted descriptively and rated as very-low-certainty results.

### 2.3. Information Sources

We searched PubMed/MEDLINE, CINAHL, Scopus, PsycINFO, and the Cochrane Library for studies published between 2000 and 31 May 2025. Reference lists of included articles and key reviews were also hand-searched. Gray literature repositories were not included by design, as this review focused on peer-reviewed journal publications. One complete search strategy (PubMed) is presented in Table S3, with database-specific adaptations available upon request from the corresponding author. The completed PRISMA 2020 checklist is provided in the Supplementary Materials (Table S1). Limiting the search to English-language publications was necessary due to reviewer language expertise; the potential for language bias is discussed in Section 4.4.

### 2.4. Search Strategy

Strategies combined controlled vocabulary (e.g., “Cleft Palate”, “Velopharyngeal Insufficiency”, “Speech Therapy”, “Telemedicine”) and keywords (e.g., telehealth, telepractice, tele; speech–language pathology; child; pediatric *), as adapted to each database. Our search was restricted to English-language publications and publication year was ≥2000.

### 2.5. Selection Process

Records were exported and de-duplicated prior to screening. Six reviewers worked in pairs to screen titles/abstracts in duplicate, followed by duplicate full-text screening against the PICOS. Disagreements were resolved through discussion or, if needed, adjudication by a seventh reviewer (supervisor). The reasons for exclusion at full text were recorded and are summarized in the PRISMA 2020 flow diagram.

### 2.6. Data Collection Process and Data Items

A piloted extraction form captured study metadata (authors, year, country/setting, design), participant characteristics (age, cleft/VPD status), intervention details (service type, delivery platform, duration/intensity, caregiver-mediated vs. clinician-led), comparator (if any), and outcomes. The following criteria were used to define effectiveness, satisfaction, and accessibility:

Effectiveness: PCC, articulation/phonology error patterns, intelligibility, resonance/nasality ratings, and other validated speech outcomes with timepoints.

Satisfaction: Caregiver/patient/clinician ratings (e.g., Likert scales) and qualitative feedback.

Accessibility: Attendance/completion, travel/time saved, technology access, and equity-relevant barriers.

Extraction was performed independently by two reviewers per study with consensus reconciliation. The extraction form was piloted on two representative studies to confirm clarity and consistency prior to full data extraction.

### 2.7. Risk-of-Bias Assessment

Randomized trials were appraised using the Cochrane Risk of Bias 2.0 (RoB 2) tool. All non-randomized designs, including cross-sectional, pre–post single-arm, and service-evaluation studies, were assessed using the relevant Critical Appraisal Skills Programme (CASP) checklists. Two reviewers independently conducted the assessments and resolved any discrepancies via consensus. Study-level judgments are summarized in Table 1 and Table 2 (CASP) and Table 3 (RoB 2). The Newcastle–Ottawa Scale (NOS) was initially considered but excluded to avoid redundancy; therefore, RoB 2 was used for the randomized trial and CASP for non-randomized/service designs.

**Table 1:** Risk-of-bias assessment using CASP criteria (Set 1).

CASP Criterion	[20]	[21]	[15]	[22]	[23]	[6]
1. Focused issue	Yes	Yes	Yes	Yes	Yes	Yes
2. Appropriate method	Yes	Yes	Yes	Yes	Yes	Yes
3. Subjects recruited acceptably	Yes	Yes	Yes	Cannot tell	Cannot tell	Yes
4. Measures accurately measured	Yes	Yes	Cannot tell	Yes	Yes	Yes
5. Data collection addressed issue	Yes	Yes	Cannot tell	Yes	Yes	Yes
6. Enough participants	No	Yes	No	Cannot tell	No	Cannot tell
7. Results presented	Yes	Yes	Cannot tell	Yes	Yes	Yes
8. Rigorous data analysis	No	Yes	No	Cannot tell	Yes	Yes
9. Clear statement of findings	Yes	Yes	Yes	Yes	Yes	Yes
10. Applicability to population	Yes	Yes	Yes	Yes	Cannot tell	Yes
11. Value of the research	Yes	Yes	Yes	Yes	Yes	Yes
Overall risk of bias	Moderate	Low–Moderate	Moderate	Moderate	Moderate–High	Low–Moderate

**Table 2:** Risk-of-bias assessment using CASP criteria (Set 2).

CASP Criterion	[16]	[24]	[25]
1. Focused issue	Yes	Yes	Yes
2. Appropriate method	Yes	Yes	Yes
3. Subjects recruited acceptably	Cannot tell	Cannot tell	Cannot tell
4. Measures accurately measured	Cannot tell	Yes	Yes
5. Data collection addressed issue	Yes	Yes	Yes
6. Enough participants	Cannot tell	Cannot tell	Cannot tell
7. Results presented	Yes	Yes	Yes
8. Rigorous data analysis	Cannot tell	Cannot tell	Yes
9. Clear statement of findings	Yes	Yes	Yes
10. Applicability to population	Yes	Yes	Yes
11. Value of the research	Yes	Yes	Yes
Overall risk of bias	Moderate	Moderate	Moderate

Note: (Table 1 and Table 2): CASP was used pragmatically for descriptive/service and single-group pre–post post-design experiments.

**Table 3:** Summary of Risk-of-Bias Assessment (RoB 2) for the Included Randomized Controlled Trial.

Domain	[26]	Risk Judgment
Randomization process	Computer-generated sequence; independent allocation	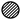 Low risk
Deviations from intended interventions	Parent-delivered therapy; no blinding; potential protocol deviations	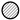 Some concerns
Missing outcome data	Minimal attrition; balanced across groups	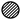 Low risk
Measurement of the outcome	Outcome assessors likely unblinded	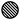 High risk
Selection of the reported results	No preregistered protocol; potential selective reporting	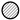 Some concerns
Overall risk of bias	Combination of minor and measurement-related concerns	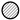 Some concerns

Legend: 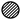 = Low risk, 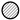 = Some concerns, 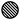 = High risk.

### 2.8. Effect Measures

Where available, we extracted between-group effect estimates (e.g., mean differences, risk ratios) or within-group pre–post changes with 95% CIs. For agreement studies (remote vs. in-person assessment), we extracted agreement coefficients (e.g., Cohen’s κ, ICC). When standardized synthesis was not possible, we coded the effect direction (benefit/no difference/harm) for the primary outcome domain. The effect direction was coded as positive, neutral, or negative relative to the primary outcome, facilitating narrative grouping in alignment with SWiM guidance [27]. The direction-of-effect results are summarizedin tabular form and described within the narrative synthesis, consistent with SWiM recommendations.

### 2.9. Synthesis Methods (SWiM)

Owing to the heterogeneity in study designs, interventions, and outcomes, a quantitative meta-analysis was not performed. Instead, the synthesis followed the SWiM framework [27]. Studies were grouped by service type (assessment, therapy, or follow-up) and by delivery model (caregiver-mediated versus clinician-led). Structured narrative summaries were produced, accompanied by effect-direction coding and tabulated outcome ranges. The direction-of-effect results are summarized in tables in Section 3.4 and described within the narrative synthesis, consistent with SWiM reporting recommendations. Sensitivity analyses were planned to exclude studies at critical risk of bias and to examine findings from non-pandemic contexts; however, these analyses could not be undertaken because of the small number and heterogeneity of included studies.

### 2.10. Reporting Bias Assessment

We compared the outcomes to protocols/registrations where available and assessed selective reporting within the RoB tools. Trial registries and authors’ statements were consulted when feasible.

### 2.11. Certainty Assessment

Certainty ratings (GRADE) are summarized narratively in the Results section; detailed rationale for downgrades is available from the authors upon request.

## 3. Results

### 3.1. Study Selection

**Figure 1:**
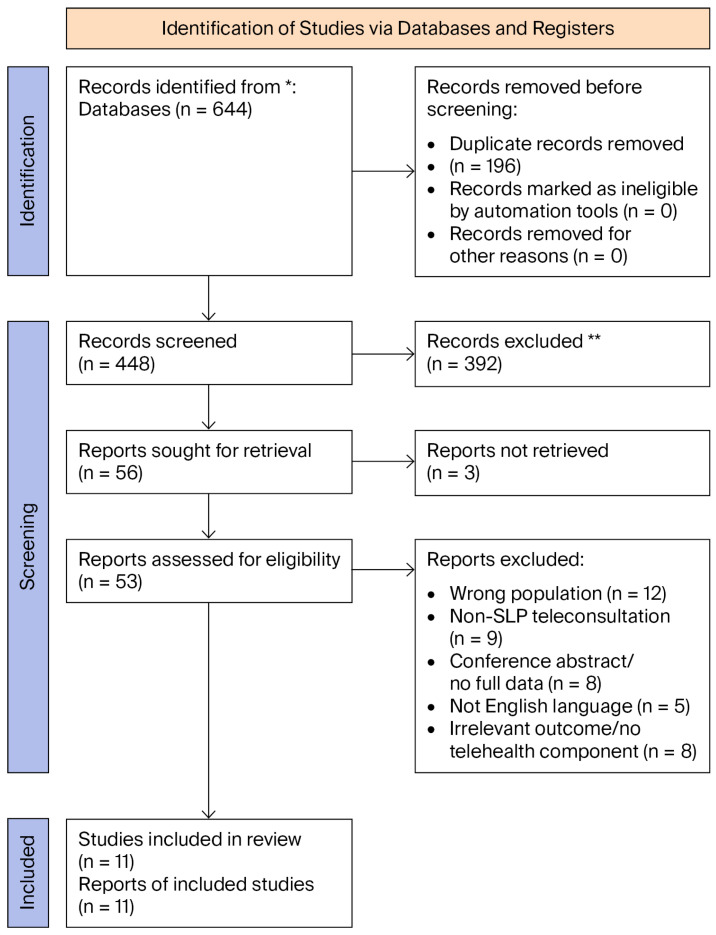
PRISMA 2020 flow diagram depicting the identification, screening, eligibility, and inclusion of studies. * = Databases searched (PubMed, CINAHL, Scopus, PsycINFO, and Cochrane Library); ** = Records excluded after title/abstract screening (not meeting inclusion criteria).

Database searches conducted across PubMed, CINAHL, Scopus, PsycINFO, and the Cochrane Library identified records that, after screening and eligibility assessment, yielded 11 studies that met the inclusion criteria for telehealth SLP in children with cleft palate/VPD (Figure 1).

### 3.2. Risk of Bias

The single randomized trial was assessed using RoB 2 [16]. Non-randomized observational and pre–post/service designs were appraised using CASP checklists. Common limitations included small samples, heterogeneous outcomes, limited blinding, and incomplete reporting of treatment dose or fidelity. Study-level judgments are presented in Table 1 and Table 2 (CASP) and Table 3 (RoB 2). Item-level worksheets are available on request.

### 3.3. Study Characteristics and Extracted Data

Across the 11 studies, populations ranged from toddlers to early adolescence; interventions spanned remote assessment, caregiver-mediated articulation/phonology therapy, clinic-led tele-blocks, and virtual MDT follow-up. Outcomes were grouped as speech effectiveness, caregiver satisfaction, and accessibility. A study-level overview appears in Table 4 (designs/samples/settings), Table 5 (interventions and main results), and Table 6 (effectiveness/satisfaction/accessibility mapping).

**Table 4:** A summary of the information and participants from the included studies.

Authors (Year)	Study Design	Sample Size	Setting	Age Range (Years)	N with CP/VPD
[20]	Pilot telepractice parent training (single-group study)	3 cleft + 1 control	USA (telepractice)	1.75–2.25	3
[21]	Pre–post intervention	43 (completed)	Mexico (Hablarte e Integrarte A.C.)	4–12 (M = 7.04, SD = 2.59)	43
[25]	Pre–post prospective study	19	Thailand (Srinagarind Hospital)	5–13	19
[22]	Observational pre–post study	16	Japan (Aichi Gakuin University Dental Hospital)	~4–12 (M = 6.4)	16
[26]	Randomized controlled trial	44	UK and Ireland	2.75–7.17	44
[6]	Cross-sectional comparative study (Remote vs. In-Person assessment)	9	Mexico and USA	5–14	9
[24]	Descriptive cross-sectional study (tele-therapy, cross-border)	8/10 completed	Baltimore, USA, and Nicaragua	3–17	8
[16]	Service evaluation (virtual MDT clinic)	N/A	N/A	N/A	N/A
[13]	Cross-sectional survey	212	UK (Cleft Collective)	0.5–12.4	212
[23]	Observational case study (telepractice)	1	Hong Kong	10	1
[15]	Service delivery report (virtual MDT; Zoom/MyChart)	N/A	USA	N/A	N/A

### 3.4. Synthesis by Outcome Domain

This study produced the following results regarding effectiveness, satisfaction, and accessibility:

Effectiveness: Most studies reported within-group improvements for caregiver-mediated articulation/phonology programs (e.g., EMT+PE; therapist-supervised, parent-led articulation) and moderate-to-high agreement for several parameters in remote speech assessment compared with in-person ratings, with weaker agreement for airflow/nasality-dependent features (see Table 5 and Table 6). Most intervention studies incorporated an initial orientation or parent-training component covering platform use and articulation cueing methods, which facilitated consistent home implementation.

Satisfaction: Where measured, caregiver satisfaction was high, citing convenience, reduced travel/time burden, and increased involvement (Table 6).

Accessibility: Studies frequently documented improved access (reduced travel/time; continuity during service disruptions), though this was tempered by digital-divide barriers and variable online engagement (Table 6). Across outcome domains, most studies demonstrated either positive within-group improvement or parity with in-person care, particularly for caregiver-mediated articulation therapy and remote assessment reliability. Effect-direction coding outcomes are embedded in Table 5 and Table 6 to indicate the overall direction of findings across outcome domains.

**Table 5:** Summary of interventions and main results.

Authors (Year)	Intervention	Main Results
[20]	EMT+PE delivered via telepractice (parent coaching)	Increased parent strategy use; gains in talking rate, speech production, and expressive vocabulary. (post-treatment follow-up only).
[21]	Group SLP via telepractice (Whole Language Model)	Significant improvement in compensatory articulation severity (*p* < 0.001). (post-treatment follow-up only).
[25]	Hybrid face-to-face and Zoom telepractice	Significant improvements in PCC, resonance, intelligibility, and acceptability; high caregiver satisfaction. (3-month follow-up).
[22]	Telepractice therapy during COVID-19	Outcomes improved or maintained; 100% parental satisfaction. (post-treatment follow-up only).
[26]	Parent-led articulation therapy via Video vs. Routine care	Both groups improved; no significant between-group differences (parity with usual care). (3-month RCT follow-up).
[6]	Real-time Remote vs. In-Person assessment	High agreement on many parameters; weaker for airflow/nasality-dependent features. (single assessment session).
[24]	Tele-SLP with interpreter support (cross-border)	Articulation gains; improved social participation/quality of life. (post-treatment follow-up only).
[16]	Virtual MDT clinic (JPRAS)	Preserved care planning/continuity; identified practical constraints. (follow-up period not specified).
[13]	Tele-SLP service during COVID-19	Most parents rated therapy as at least “somewhat effective”; tech/engagement issues noted. (service-period survey).
[23]	Telepractice articulation therapy (VPD; Cantonese)	Positive speech production changes with strong parent involvement. (post-treatment follow-up only).
[15]	Virtual MDT (Zoom/MyChart)	Enabled flexible coordination; reduced travel needs. (follow-up period not specified).

**Table 6:** Overview of effectiveness, satisfaction, and accessibility.

Authors (Year)	Effectiveness	Satisfaction	Accessibility
[20]	Improved speech/language via caregiver-mediated model (post-treatment follow-up only).	Not explicitly reported	Feasible for toddlers at home
[21]	Significant articulation improvements (post-treatment follow-up only).	Not reported	High; group telepractice is workable
[25]	Improved PCC/resonance/intelligibility (3-month follow-up).	Very high caregiver satisfaction	High; reduced costs/burden
[22]	Maintained/improved therapy outcomes (post-treatment follow-up only).	100% parental satisfaction	High for families with internet/video
[26]	Telehealth ≈ routine care on primary outcomes (3-month RCT follow-up).	Positive feedback in feasibility phase	Video platform bridged gaps
[6]	Remote ≈ in-person for many perceptual ratings (single assessment session).	Positive parent feedback	Cross-border evaluation feasible
[24]	Articulation gains; QOL improvements (post-treatment follow-up only).	High satisfaction (parent-reported)	Interpreter-supported cross-border model
[16]	MDT continuity: SLP components preserved (follow-up period not specified).	Not formally measured	Reduced travel; flexible planning
[13]	Most rated therapy “somewhat/very effective” (service-period survey).	Generally positive; engagement/tech issues	Access dependent on connectivity
[23]	Positive articulation changes in case study (post-treatment follow-up only).	Inferred high (family engagement)	Effective despite constraints
[15]	MDT coordination maintained (follow-up period not specified).	Provider-reported positives	Enabled access for remote families

Notes (Table 4, Table 5 and Table 6): PCC = percent consonants correct; MDT = multidisciplinary team; QOL = quality of life.

## 4. Discussion

This systematic review found early but limited evidence suggesting that telehealth may offer a feasible and acceptable means of delivering SLP services for children with cleft palate and VPD. However, the certainty of this evidence is rated as very low to low due to concerns regarding the use of small samples, heterogeneous designs, and risk of bias; thus, the conclusions should be interpreted cautiously.

### 4.1. Effectiveness of Telehealth Interventions

Across the 11 included studies, telehealth for children with cleft palate and/or VPD was feasible and generally beneficial for speech outcomes when interventions were well defined, and family engagement was high. Caregiver-mediated articulation/phonology programs (e.g., EMT+PE; therapist-supervised, parent-led articulation) consistently produced within-group gains, such as improvements in percent consonants correct (PCC) and reductions in compensatory errors, in small, single-center studies [20,21,22,25]. Across the reviewed studies, telehealth appeared particularly effective for toddlers and preschool-aged children when parents were coached to implement therapy strategies at home. In contrast, sustaining motivation and attention during online sessions posed greater challenges for school-aged participants, consistent with developmental differences in self-regulation and online engagement. The single randomized trial reported no significant differences between parent-led telehealth and routine care on primary speech/participation outcomes, which is consistent with parity rather than inferiority [26]. Where studies lacked an in-person comparator, improvements were interpreted as within-group trends rather than as evidence of superiority, in keeping with the mixed-design nature of the available literature.

Remote assessment demonstrated moderate-to-high agreement with in-person ratings for several perceptual parameters, with weaker agreement for airflow/nasality-dependent features [6]. Interventional tele-SLP in cross-border contexts also reported articulation gains alongside improved social participation [24]. Virtual multidisciplinary (MDT) models preserved the continuity of cleft care, with SLP components being maintained during service disruptions [15,16].

Effectiveness varied with age, severity, and technology reliability; in younger children, caregiver training/coaching was pivotal, whereas in older children, online engagement and sustained attention could be rate-limiting [20,22]. Across therapy models, the reporting of dose (session length/number), treatment fidelity, and standardized outcome instruments was inconsistent, limiting cross-study comparisons.

Virtual multidisciplinary team (MDT) clinics, integrating SLP input with surgical and psychosocial follow-up, preserved the collaborative benefits of in-person cleft care and were perceived by families as enhancing continuity and comprehensive management. Detailed certainty ratings for each outcome domain, based on the GRADE approach, are provided in the Supplementary Materials (Table S2).

### 4.2. Satisfaction with Telehealth

Where measured, caregiver satisfaction was high, with convenience, reduced travel/time costs, and greater involvement in therapy cited as reasons for high approval [15,22,25]. Parents also reported comfort with real-time remote assessments [6]. Some families initially viewed telehealth as a pandemic workaround but later acknowledged scheduling advantages and reduced burdens [13]. Measurement tools were usually non-standardized (ad hoc Likert items or service evaluations), so effect sizes and between-study comparisons are uncertain; satisfaction should be interpreted as convergent but low-certainty evidence of acceptability.

### 4.3. Accessibility and Equity

Telehealth improved access by minimizing travel burdens and enabling continuity in multidisciplinary follow-up, particularly during periods of disrupted in-person care [15,16,25]. Caregiver-reported time/cost savings were notable where captured [25]. At the same time, digital-divide barriers (bandwidth, device availability, digital literacy) and child engagement online were recurrent constraints [13,28], reflecting broader disparities in digital access and telehealth use reported across healthcare systems [18,29]. Interpreter-supported and cross-border models illustrate how telehealth can address geographic and linguistic barriers [6,24]. Broader cleft telehealth programs, though excluded from this synthesis due to non-SLP outcomes, document substantial travel reductions and reliable peri-/postoperative care [14] and low-cost technology pathways suitable for remote evaluations [30], triangulating SLP-specific accessibility findings.

### 4.4. Strengths and Limitations of This Review

The strengths of this review include its adherence to PRISMA 2020, the use of RoB 2 for the RCT and CASP for non-randomized and service studies, and the inclusion of pre–post studies, SWiM-aligned narrative synthesis, and GRADE certainty ratings. The dataset spans multiple regions and service configurations (assessment, therapy, MDT follow-up), enhancing external relevance (e.g., the USA, the UK/Ireland, Mexico, Thailand, Japan, Hong Kong).

This review’s limitations reflect those of the underlying evidence: small samples; heterogeneity in interventions and outcomes; short follow-up; predominantly non-randomized designs at serious risk of bias (confounding, outcome measurement, absence of concurrent comparators). Satisfaction instruments were non-standardized. Inclusion was restricted to English-language, peer-reviewed reports published from 2000 onward, which may have introduced language and publication bias. Given these constraints, the overall certainty of evidence was very low to low across domains.

### 4.5. Implications for Practice and Research

Clinical Implications:

- Telehealth can be a reasonable first-line or adjunct delivery mode for articulation and phonology therapy in pediatric cleft/VPD, especially when caregiver-mediated with SLP coaching and when travel burden is high [20,21,25,26].
- Professionals should apply telehealth strategically for triage, caregiver coaching, and routine follow-up within multidisciplinary (MDT) pathways; targeted in-person sessions remain essential for complex perceptual or instrumental assessments (e.g., resonance or airflow evaluation) [6].
- Standardization of documentation, treatment fidelity, and core outcomes (e.g., PCC, validated perceptual scales) will strengthen quality assurance and future evidence synthesis [16,22].
- Equity must be addressed proactively through low-bandwidth options, interpreter services, and culturally responsive materials to ensure inclusive access [12,20].

Research Implications:

Future investigations should prioritize:

(i) Adequately powered comparative trials with standardized and blinded outcome assessment;
(ii) Validation of remote measures for resonance and nasality, including acoustic or automated approaches;
(iii) Implementation and dose–response studies to define minimum effective telehealth intensity;
(iv) Cost-effectiveness and digital equity evaluations;
(v) Long-term follow-up examining participation and quality-of-life outcomes and the use of multicentre randomized controlled designs with standardized outcome sets to strengthen external validity and durability of findings.

The findings of this review align with and extend the broader telehealth literature in speech-language pathology. Prior scoping and narrative reviews in mixed pediatric populations have concluded that telehealth is generally feasible and acceptable, with clinical outcomes often comparable to in-person therapy when caregiver engagement is high [10,11,31]. Within the cleft/VPD subset, the present synthesis shows a similar pattern: caregiver-mediated articulation and phonology models yield within-group gains, and the sole RCT demonstrated parity with conventional care [20,21,26].

On assessment, the observed moderate-to-high agreement between remote and in-person ratings for most perceptual parameters [6] mirrors broader SLP telehealth findings. At the same time, resonance and airflow judgments remain best suited to in-person evaluation [9,32]. Evidence from virtual multidisciplinary clinics during the COVID-19 pandemic [15,16] further corroborates accessibility benefits documented in non-SLP telehealth contexts [14,33]. Conversely, studies from lower-resource settings [28] underscore that equitable telehealth implementation must address barriers such as connectivity, device access, and digital literacy.

## 5. Conclusions

Telehealth shows preliminary promise for improving accessibility and caregiver involvement in speech–language pathology services for children with cleft palate and VPD. The current evidence remains limited by the predominance of small, heterogeneous studies and low methodological certainty; therefore, these findings should be viewed as provisional. Continued high-quality research, including adequately powered comparative trials, validated remote assessment tools, and equity-focused implementation studies, is needed to establish the effectiveness, sustainability, and best practices for telehealth within interdisciplinary cleft care. Socioeconomic disparities and infrastructure variability can significantly impact the feasibility and adoption of telehealth. Addressing these “digital divide” factors, through affordable internet access, culturally appropriate materials, and targeted caregiver training, is essential for equitable implementation.

Despite the low–very-low certainty of evidence, telehealth represents a feasible adjunct to traditional in-person care for children with cleft palate and velopharyngeal dysfunction and should be integrated thoughtfully within hybrid service pathways.

## Data Availability

All data extracted for this review (extraction tables, item-level risk-of-bias worksheets, and GRADE notes) are available from the corresponding author on reasonable request.

## Supplementary Materials

The following supporting information can be downloaded at: https://www.mdpi.com/article/10.3390/children12111523/s1, Table S1: PRISMA 2020 checklist; Table S2: GRADE Summary of Findings; Table S3: detailed PubMed search strategy.
