# Supplementary material for: Telehealth Delivery of Speech–Language Pathology Services for Children with Cleft Palate and Velopharyngeal Dysfunction: A Systematic Review
Source: Children (Basel). 2025 Nov 11;12(11):1523. doi: 10.3390/children12111523 (PMC12651934; doi:10.3390/children12111523)
Supplement: Supplementary file 1 [file children-12-01523-s001.zip › children-3902488-Tables S1 and S2.pdf]

Supplementary Materials list

- **Table S1.** PRISMA 2020 checklist.
- **Table S2.** GRADE Summary of Findings.

Table S1. PRISMA 2020 checklist for this systematic review, with page and section references indicating where each item is addressed in the manuscript.

Section/Topic	Item #	Checklist item	Reported location in manuscript
**TITLE**	1	Identify the report as a systematic review.	Title page ("A PRISMA-Compliant Systematic Review")
**ABSTRACT**	2	Provide a structured abstract (Background, Methods, Results, Conclusions).	Structured Abstract
**INTRODUCTION**	3	Rationale: describe the rationale in the context of what is known.	Introduction, 1–3
	4	Objectives: explicit questions with reference to PICOS.	Introduction, final
**METHODS**	5	Eligibility criteria (PICOS, study designs, exclusions).	2.2 Eligibility Criteria
	6	Information sources (databases, date of last search).	2.3 Information Sources
	7	Search strategy (full strategies, limits).	2.4 Search Strategy (+ Supplementary Methods if provided)
	8	Selection process (screening in duplicate, conflict resolution).	2.5 Selection Process
	9	Data collection process (piloted forms, duplicate extraction).	2.6 Data Collection Process
	10a	Data items: outcomes and variables extracted.	2.6 Data Items
	10b	Other variables sought (e.g., participant/intervention characteristics, setting, funding) and assumptions about missing/unclear information.	2.6 Data Items
	11	Risk-of-bias assessment methods (tools, reviewers, process).	2.7 Risk-of-Bias Assessment
	12	Effect measures specified for each outcome domain.	2.8 Effect Measures

Section/Topic	Item #	Checklist item	Reported location in manuscript
	13a	Synthesis methods: deciding which studies entered each synthesis.	2.9 Synthesis Methods (SWiM)
	13b	Methods to handle data/prepare for synthesis.	2.9
	13c	Methods to tabulate or visually display results.	Tables 4–6 (Figure 1 for flow)
	13d	Methods to combine results; rationale for narrative synthesis (no meta-analysis).	2.9
	13e	Methods to explore causes of heterogeneity.	2.9 (planned; limited by heterogeneity)
	13f	Sensitivity analyses (planned/undertaken).	2.9 (planned; none feasible)
	14	Reporting bias assessment (planned approach).	2.10 Reporting Bias Assessment
	15	Certainty assessment (GRADE).	2.11 Certainty Assessment
**RESULTS**	16a	Study selection: numbers screened, assessed, included.	3.1 Study Selection; Figure 1
	16b	Reasons for exclusions at full text.	3.1; Figure 1
	17	Study characteristics.	3.3; Table 4
	18	Risk of bias in studies.	3.2; Tables 1–3
	19	Results of individual studies.	3.4; Tables 5–6
	20a	Results of syntheses (overall findings by grouping).	3.4; 4.1–4.3
	20b	Results of statistical syntheses (meta-analysis), incl. effect sizes and uncertainty.	Not applicable (no meta-analysis)
	20c	Results of investigations of heterogeneity.	Not applicable / limited (see 2.9)
	20d	Results of sensitivity analyses.	Not applicable / none feasible (see 2.9)
	21	Reporting biases: risk of bias due to missing results across studies.	3.2 (and/or 3.4); 4.4 Limitations
	22	Certainty of evidence for each outcome domain.	2.11; 4.4; Supplementary GRADE Summary Table
**DISCUSSION**	23a	General interpretation of results.	4.1–4.3
	23b	Limitations of the evidence included.	4.4

Section/Topic	Item #	Checklist item	Reported location in manuscript
	23c	Limitations of the review processes.	4.4
	23d	Implications for practice, policy, and research.	4.5–4.7
**OTHER INFORMATION**	24a	Registration and protocol.	2.1 (not prospectively registered)
	24b	Protocol amendments/deviations.	2.1 (deviations described)
	25	Support/funding.	Back matter: Funding
	26	Competing interests.	Back matter: Conflicts of Interest
	27	Availability of data, code, and materials.	Back matter: Data Availability

Table S2. GRADE Summary of Findings for telehealth SLP in pediatric cleft/VPD across three outcome domains (effectiveness, caregiver satisfaction, accessibility). Domains assessed: risk of bias, inconsistency, indirectness, imprecision, publication bias

Outcome domain	Risk of bias	Inconsistency	Indirectness	Imprecision	Publication bias	Overall certainty (GRADE)	Brief rationale
Effectiveness (speech outcomes)	Serious	Serious	Not serious	Serious	Suspected	Low	Evidence dominated by small, single-center pre–post designs; one RCT with some concerns; heterogeneous interventions/outcomes; short follow-up; limited precision.
Caregiver satisfaction	Serious	Serious	Not serious	Serious	Suspected	Very low	Mostly non-validated Likert/service evaluations; varying measures and contexts; small samples; wide uncertainty around estimates.
Accessibility (attendance, travel/time saved, feasibility)	Serious	Serious	Not serious	Serious	Suspected	Very low	Descriptive service reports; benefits consistent but imprecise; potential selective reporting; limited comparative data.
